# Ketogenic diet with oxyresveratrol and zinc inhibits glioblastoma and restores memory function and motor coordination

**DOI:** 10.32604/or.2024.049538

**Published:** 2025-01-16

**Authors:** TANVI VIJAY GUJARAN, VIGNESH BALAJI EASWARAN, RUNALI SANKHE, PUGAZHANDHI BAKTHAVATCHALAM, HERMAN SUNIL DSOUZA, K. SREEDHARA RANGANATH PAI

**Affiliations:** 1 Department of Pharmacology, Manipal College of Pharmaceutical Sciences, Manipal Academy of Higher Education, Manipal, 576104, India; 2 Division of Anatomy, Department of Basic Medical Science, Manipal Academy of Higher Education, Manipal, 576104, India; 3 Department of Anatomy and Physiology, American University of Antigua, University Park, Antigua, W1451, West Indies; 4 Department of Radiation Biology and Toxicology, Manipal School of Life Sciences, Manipal Academy of Higher Education, Manipal, 576104, India

**Keywords:** Glioblastoma (GBM), Ketogenic diet (KD), Oxyresveratrol, Zinc, Warburg effect

## Abstract

**Background:**

To date, there is no effective cure for the highly malignant brain tumor glioblastoma (GBM). GBM is the most common, aggressive central nervous system tumor (CNS). It commonly originates in glial cells such as microglia, oligodendroglia, astrocytes, or subpopulations of cancer stem cells (CSCs). Glucose plays an important role in the, which energy metabolism of normal and cancer cells, but cancer cells exhibit an increased demand for glucose is required for their differentiation and proliferation. The main aim of this study is to explore the anti-cancer efficacy of the ketogenic diet against GBM. Also, this research focuses on the identification of the catalytic action of zinc in epigenetic modulators such as oxyresveratrol and ensures the combinatorial effect in the treatment of GBM.

**Method:**

In this study, we have evaluated various parameters to understand the therapeutic efficacy of the treatment groups through *in vivo* experiments against aggressive brain tumors. Intracerebroventricular experiments were performed to induce the tumor in the animals and estimate the tumor burden and proliferative index. Followed by the Morris water maze, an open field test, and rota rod was performed to evaluate the memory and motor coordination. To understand the glucose, and ketone level modification before and after treatment, the level of glucose and ketone was analyzed. Moreover, the zinc level is assessed using flame atomic absorption spectroscopy.

**Results:**

The results suggested that the ketogenic diet has an anti-cancer efficacy against C6-induced GBM cell lines. Also, it exerts a synergistic effect with the epigenetic modulator, oxyresveratrol, and zinc against GBM cell lines. Moreover, the treatment groups improved memory and motor coordination and modified the glucose and ketone levels to reduce the tumor burden and Ki-67 proliferative index.

**Conclusion:**

This study revealed the therapeutic effect of the ketogenic diet along with its combination such as oxyresveratrol and zinc against the C6-induced GBM in the Wistar rats. Also, it improved memory and motor coordination and reduced tumor growth. It also modified the glucose and ketone levels in the tumor-induced animal and supported to diminish the tumor burden.

## Introduction

Glioblastoma (GBM) is the most common, aggressive primary tumor of the central nervous system (CNS), and it is considered the most difficult form of brain cancer to treat. GBM is associated with a high rate of death in people with a primary brain tumor [1–3]. In the US, between 2014 and 2018, approximately 83,029 deaths were reported due to GBM and other CNS tumors among the adult population, and in 2021, 25,690 adults were diagnosed with GBM [4]. In India, approximately 5 to 10 cases per 100,000 people are diagnosed with CNS tumors [5]. GBM mostly originates either from glial cells, such as astrocytes, microglia, oligodendrocytes, and ependymal cells, or from a subpopulation of cancer stem cells (CSCs) residing in the tissue. Memory impairment, weakness, or numbness in the hands and legs; headache; speech problems; and seizures are complications that can further worsen patients with GBM [6]. GBM is the 3^rd^ most common cause of death in patients between the ages of 15 and 34 years, especially since 2.5% of the deaths occur due to malignant glioma [6]. GBM is classified as a grade 4 astrocytoma by the World Health Organization (WHO) [7] and is the most aggressive form with a high mortality rate. Standard therapy includes surgical resection of the tumor followed by radiation and temozolomide-based chemotherapy [8]. Despite advancements in treatment paradigms such as the combination of surgical resection, radiation, and chemotherapy, the overall survival (OS) period of patients is approximately 15–18 months after diagnosis [9–11]. Although currently available treatments such as radiotherapy and chemotherapy have been shown to increase progression-free survival (PFS), the OS of GBM patients remains very poor compared with that of patients with other cancers, such as lung and breast cancer [12–14]. GBMs are classified into primary gliomas and secondary gliomas. Primary GBM differs from secondary GBM based on the genetic profile of the isocitrate dehydrogenase 1 (IDH 1) mutation. IDH1 mutation is evident in secondary GBM but not in primary GBM [15]. IDH mutations are found in approximately 80% of low-grade gliomas [16]. One of the major challenges faced in treating GBM is the presence of the blood‒brain barrier (BBB) [17]. Another major reason for treatment failure is the development of resistance by the primary tumor to chemotherapeutic agents by enhancing the efflux mechanism and direct inactivation of the drug [18].

Often, there is an alteration in the metabolism of cancer cells compared with that of normal healthy cells; this phenomenon is called the “Warburg effect” [19] and, in other words, aerobic glycolysis [20]. The differentiation and proliferation of tumor cells are highly dependent on glucose [20]. Increased glucose uptake not only results in the synthesis of ATP but also the synthesis of fatty acids and nucleic acids, which are further utilized for cell maintenance [21]. It leads to the rapid proliferation of cells, and this process can occur even in a hypoxic state as well as in dysfunctional mitochondria [22]. Since glucose and glutamine are the two major fuels required by GBM cells for Where is reference 22 between 21 and 23 their growth and proliferation, these fuels are considered targets in the therapeutic management of GBM [23]. Hence, diet-based therapy, which affects cancer cell metabolic pathways, is called “ketogenic diet” therapy [23,24]. A ketogenic diet (KD) is a high-fat (approximately 90%) low-carbohydrate diet with adequate amounts of protein [25] and thus results in increased ketone body production. These ketone bodies act as alternative sources of energy and are transported to many organs, including the brain. It is the only source of energy for the brain when the supply of glucose is limited. However, cancer cells cannot survive on ketone bodies due to metabolic inflexibility, thus resulting in cancer cell death [21]. Under normal conditions, a healthy brain is dependent mainly on glucose and obtains energy through the complete oxidation of glucose. It produces pyruvate, which is further converted into acetyl-CoA and enters the TCA cycle to support the electron transport chain. Thus, glycolysis and respiration remain interlinked, resulting in the efficient production of ATP along with lactic acid [26,27]. However, during conditions such as fasting or a low-carbohydrate diet, ketone bodies are utilized as an alternative source of energy. Ketone bodies can cross the BBB through the transporter monocarboxylate transporter (MCT), which is located in astroglia and endothelial cells [28]. When a high-carbohydrate diet is replaced with a KD, this condition will create a starvation-like environment in which ketone bodies are utilized by normal brain cells, whereas cancer cells are glucose-dependent in nature. Henceforth, tumor cells cannot consume ketone bodies as an alternative fuel and exert their anticancer effects [29–33]. To date, studies have proven the safety and efficacy of a KD in humans [34]. In addition, an *in vivo* study also showed the ability of a KD to inhibit tumor growth and increase the OS of patients [13]. Thus, recent studies have demonstrated that KD therapy, as an alternative approach or combination therapy combined with radiation and chemotherapy, inhibits the molecular pathway of angiogenesis, thereby attenuating the growth of malignant glioma cells [13,35].

Oxyresveratrol, a derivative of resveratrol [36], is a naturally occurring phytochemical that is abundant in peanuts, grapes, and red wine. It has been found to have anticancer effects on various cancer cell lines [37]. Natural compounds have been shown to restrict tumor growth and thus induce GBM cell death [38]. The anticancer effect of oxyresveratrol is regulated by the inhibition of cancer cell proliferation, neuroinflammation, angiogenesis, and metastasis. Additionally, by inducing autophagy, various therapeutic actions against tumor cells, including cell cycle arrest, interference with tumor metabolism, and upregulation of apoptosis, are promoted by oxyresveratrol [38]. Resveratrol also enhances the action of other chemotherapeutic agents, such as TMZ and paclitaxel, against GBM and other cancers of the breast, lung, and ovary [37,39]. Although resveratrol has been shown to have anticancer effects, its application is limited due to its poor bioavailability. Oxyresveratrol has better bioavailability, good water solubility [40], greater tissue permeability, and stronger scavenging ability [41] than resveratrol and thus has better anticancer activity [42]. To the best of our knowledge, no studies have demonstrated the combination of KD with epigenetic modulators such as HDAC inhibitors such as oxyresveratrol in animal models of GBM.

Zinc is an essential micronutrient found to have a cancer-preventive role. Zinc deficiency leads to abnormal cell proliferation and promotes tumor development [43]. However, the exact mechanism that results in cancer initiation and progression due to zinc dyshomeostasis is not fully understood [44,45]. Some studies have shown that changes in zinc signalling pathways and transporters are responsible for tumor development. Zinc is also involved in phosphorylation-dependent signalling cascades, such as those involving Akt and MAPKs, which regulate cell development, proliferation, and cell death [44,45]. Hence, these pathways support tumor progression due to aberrant zinc levels and thus lead to the proliferation of cancer cells and metastasis [44,45]. Zinc is an important cofactor for various proteins that are involved in crucial cellular processes, such as cellular differentiation, proliferation, and apoptosis [46], and was found to have antioxidant effects, thus providing protection against the development of brain tumors [47,48]. GBM is associated with a decrease in zinc levels, which further enhances tumor growth and thus worsens cancer conditions. Hence, zinc therapy initiates the apoptosis pathway and results in cancer cell death [43]. To date, the modulatory effect of zinc on HDAC inhibitors such as oxyresveratrol has not been studied. In our study, we evaluated the therapeutic effects of a KD, oxyresveratrol, and their combination with epigenetic modulators such as zinc on GBM and associated memory function and motor coordination.

## Materials and Methods

### Cell culture

C6 cells were used to induce GBM in female Wistar rats. Rat C6 glioma cell lines were procured from the National Centre for Cell Science (NCCS), Pune, India. C6 cancer cell lines were cultured in Dulbecco’s modified Eagle’s medium (DMEM) (Gibco Life Technologies, Thermo Scientific, Bangalore, India) supplemented with 10% fetal bovine serum (FBS) (Gibco Life Technologies, Thermo Scientific), trypsin, and penicillin-streptomycin. The cells were incubated at 37°C and 5% CO_2_.

### Animals

Female 10-week-old Wistar rats (8 animals in each group) weighing 200–250 g were procured from the Central Animal Research Facility of MAHE, Manipal. The animals were acclimated by maintaining a temperature of 23°C ± 2°C and humidity of 50% ± 5% for seven days before starting the experiment for their adjustment to a new environment. The protocol was framed according to the guidelines provided by the Committee for Control and Supervision of Experiments on Animals (CCSEA), Government of India. All animal experiments were approved by Institutional Animal Ethics Committee (IAEC), Kasturba Medical College (KMC), MAHE, Manipal (Approval Number: IAEC/KMC/117/2022).

### In vivo experiments

Animals were randomized into 7 groups (n = 8) based on the data obtained by performing the Morris water maze (MWM). MWM experiment performed to understand the leaning and memory of the animals. In MWM, the rodents will be placed in the opaque pool of water. The rodents must remember and learn to travel to the hidden platform after the training period. Animals were grouped into the following groups: normal control, sham control, disease control, ketogenic diet (KD) (fat, 80 g; carbohydrate, 16 g; and protein, 3 g), oxyresveratrol, KD + oxyresveratrol, and KD + oxyresveratrol + zinc groups. Stereotaxic intracerebral ventricular (ICV) surgery was performed to induce GBM in female Wistar rats using the C6 cell line in all the groups, excluding the normal and sham controls. From day 0, ICV surgery was performed until day 7, and tumor growth was monitored. During these 7 days, all the groups of animals were given a standard laboratory diet (*ad libitum*). From the 7^th^ to 21^st^ days, the standard laboratory diet was replaced with a KD diet for the KD group, KD + oxyresveratrol group, and KD + oxyresveratrol + zinc group. Oxyresveratrol (0.1 mL/mg, i.e., 0.2 mg/kg in 0.5% carboxymethyl cellulose (CMC)) via oral gavage was administered for 2 weeks (from day 7 to day 21). Zinc sulfate (0.4 mL) of zinc (6.9 mg/mL, i.e., 231 mg Zn/kg/diet as ZnSO4.7H2O) [49] via oral gavage was administered 1 h before the administration of oxyresveratrol to the KD + oxyresveratrol + zinc group. Blood glucose and ketone levels were monitored weekly once. After three weeks of tumor implantation, the Morris water maze (MWM), open field test (OFT), and rotarod test were performed. All the animals were humanely euthanized, brain samples were removed, and tumors were collected. The zinc level in the blood was estimated using flame atomic absorption spectrophotometry.

### Tumor induction

GBM was induced in C6 cell lines (10^5^ cells/10 µL) via intracerebroventricular injection. Animals were weighed and anesthetized using ketamine (80 mg/kg) and xylazine (10 mg/kg) via i.p. administration and placed on a stereotactic apparatus. The hair present in the head region was completely removed using hair removal cream. An incision was made from the midline of the head using a scalpel, and the bregma was exposed. Once the bregma was identified, the coordinates were adjusted to AP: 0.36 mm, ML: 3.6 mm, and DV: 5 mm from the bregma. After C6 GBM cell administration, the syringe was kept undisturbed for approximately 2 min to prevent diffusion and removed carefully. The burred hole on the skull was sealed using dental cement, a skin incision was sutured, and betadine was applied to prevent infections. Postoperatively, the animals were monitored and housed in separate cages.

### The Morris water maze was used to assess memory function

The Morris water maze (MWM) is a pool 160 cm in diameter in which 3/4^th^ of the pool is filled with water. The temperature of the water should be maintained below the body temperature of the animal. The pool was divided into four quadrants, and in one of the quadrants, a platform was placed for the animals to escape. The water was made opaque using milk powder to hide the platform. All the animals were trained for four days, i.e., an acquisition trial to find the hidden platform. On the final day, a fifth-day retention trial was performed where the animals were placed in the tank for 60 s, and their movements were recorded. The movement of all the animals was captured using a camera, and the data were analyzed using Anymaze software. Parameters such as escape latency to reach the platform, path efficiency, average speed, and total zone entries were calculated using software [6].

### Behavioral studies assessing motor function

#### Rotarod

To assess motor coordination in C6-induced GBM rats, a rotarod test was performed. This test was performed before inducing GBM on all the rats to train the animals. Before the actual study, the animals were trained to walk on the rotarod for 3 consecutive days by gradually increasing the speed from 5 rpm (rotation per minute) to 15 rpm and then on the 7^th^, 14^th,^ and 21^st^ days of the study period. The apparatus consisted of a horizontally rotating rod 8 cm in diameter. To divide the rod into equal compartments to simultaneously place multiple rats at the same time, circular separators were placed in the middle of the rod. The rats were then placed on a rotating rod at 15 rpm. When the rat fell on the rotarod platform, the photosensitive switch was tripped, and the timer for that particular compartment was stopped. Hence, the time spent by the rat on the rotating rod was recorded [50].

#### Open field test

The open field test (OFT) was performed to assess locomotor function in C6-induced GBM animals. A wooden chamber was used to place the rats individually, which was divided into 9 quadrants of 10 cm × 10 cm each. Animals were placed at the center of the chamber for 10 min. A weak light source was maintained in the experimental area. The activities of the rats were recorded by a camera placed above the chamber. After each experiment, the chamber was cleaned using 70% alcohol. Parameters such as the number of line crossings, time spent in the center square, grooming, and rearing were evaluated [51].

### Blood glucose and ketone levels

To stabilize the blood glucose levels, all the animals were fasted for 2 h before blood collection. Blood was collected by puncturing the tail vein. Blood glucose and ketone levels were estimated using freeStyle Optium Neo; Abbott. Whole blood (0.5–1.5 µL) was collected from the tail vein and placed on a glucose and ketone estimation strip. This strip was inserted into the meter to obtain the glucose (in mg/dL) and ketone (in mmol/L) levels [51].

### Zinc estimation

Blood was collected (approximately 1 mL) through the retro-orbital route in a tube containing EDTA and centrifuged at 1000 rpm and 4°C for 10 min. The plasma was then separated and used for the estimation of zinc concentration by flame atomic absorption spectrophotometry (model AA240) [52].

### Tumor volume

On the 21^st^ day after tumor implantation, all the rats were humanely euthanized, and the brains were isolated. Tumor growth was measured, and tumor volume was measured using the formula V = ½ (length × width^2^) [53].

### Tissue collection, examination (H*&*E) staining, and immunohistochemistry

Brain samples were treated with ethanol followed by xylene. Tissue impregnation was performed using paraffin wax, followed by tissue embedding and sectioning. Five-micron-thick sectioned tissue was stained with hematoxylin and eosin (H&E) and visualized under a confocal microscope at 10× and 40×. The thickness of the tissue used for the tissue examination was 5 µm.

For IHC, the total number of degenerated cells and the expression of Ki-67 (Cell Signaling Technology), a cell proliferation marker, were assessed. First, brain tissues 2.5 µm thick were mounted and treated with 0.5% or 1% H_2_O_2_ in PBS for 30 or 60 min, respectively. This procedure blocks endogenous peroxidase activity. Furthermore, the sections were incubated for 45 min in the presence of a blocking solution containing PBS, 0.4% fish skin gelatin, and 0.2% Triton X-100. The primary antibody (1:1000) was incubated with the sample for 30 min at room temperature, followed by incubation with the secondary antibody (1:1000). The levels of proliferative markers and the total number of degenerated cells in the sample were calculated under a confocal microscope at 400×. The thickness of the tissue used for the IHC was 5 µm.

### Statistical analysis

All the statistical analyses were performed using GraphPad Prism 8.0 software, and the data are expressed as the mean ± SEM. One-way ANOVA was carried out along with Dunnett’s multiple comparisons test.

## Results

### Effect of the KD and its combination of oxyresveratrol and zinc on memory in tumor-bearing animals in the Morris water maze test

**Figure 1:**
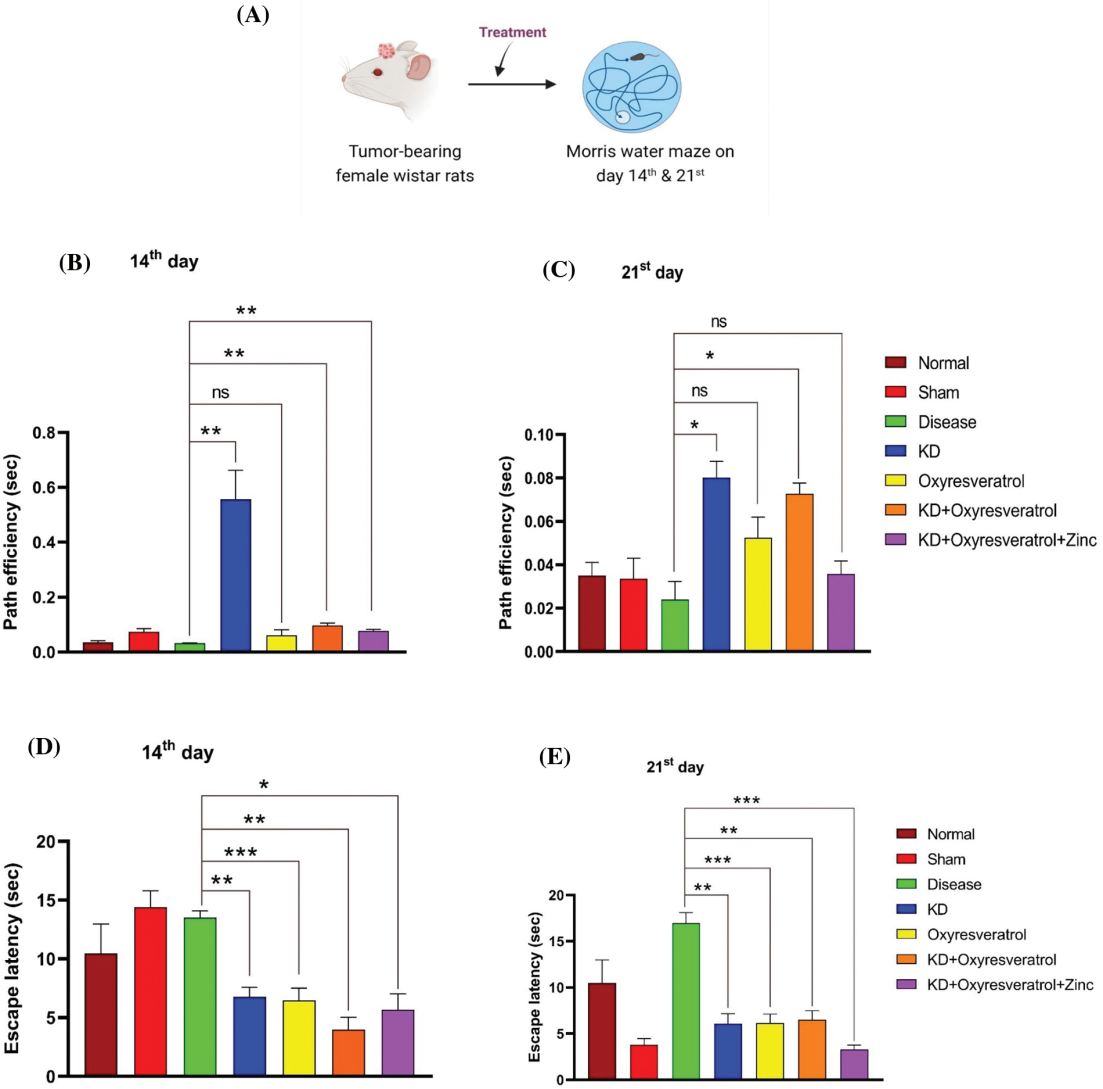
Assessment of memory function in animals using the Morris water maze (MWM). (A) Schematic representation showing the treatment of the animals and the performance of the MWM test on days 14 and 21. (B and C) Path efficiency on the 14^th^ and 21^st^ days and (D and E) escape latency on the 14^th^ and 21^st^ days. The values are expressed as the means ± SEMs. Statistical analysis was performed using one-way ANOVA with Dunnett’s multiple comparisons test. *p < 0.05, **p < 0.01, ***p < 0.001 compared to the disease control group.

A Morris water maze experiment was performed to evaluate the memory function of the animals (Fig. 1A). On the 14^th^ day, a decrease in the path efficiency of disease control was observed in comparison to that in the treatment groups. There was a significant increase in the path efficiency of the group treated with KD followed by KD + oxyresveratrol or KD + oxyresveratrol + zinc compared with that of the disease group (Fig. 1B). On the 21^st^ day, a significant increase in path efficiency was observed in the KD, KD + oxyresveratrol, and KD + oxyresveratrol + zinc groups (Fig. 1C). Compared with the normal control group, the disease control group exhibited an increase in escape latency. On the 14^th^ day, the escape latency of the disease control group was greater than that of the normal control group and all treatment groups. A significant decrease in escape latency was observed in the groups treated with KD, oxyresveratrol, KD + oxyresveratrol, and KD + oxyresveratrol + zinc compared with the disease control group (Fig. 1D). At the end of the 21^st^ day of the study period, all the treatment groups showed a significant decrease in escape latency compared with that of the disease control group (Fig. 1E).

### Effect of the ketogenic diet and its combination with oxyresveratrol and zinc on locomotor activity in tumor-bearing animals

#### Open-field test (OFT)

**Figure 2:**
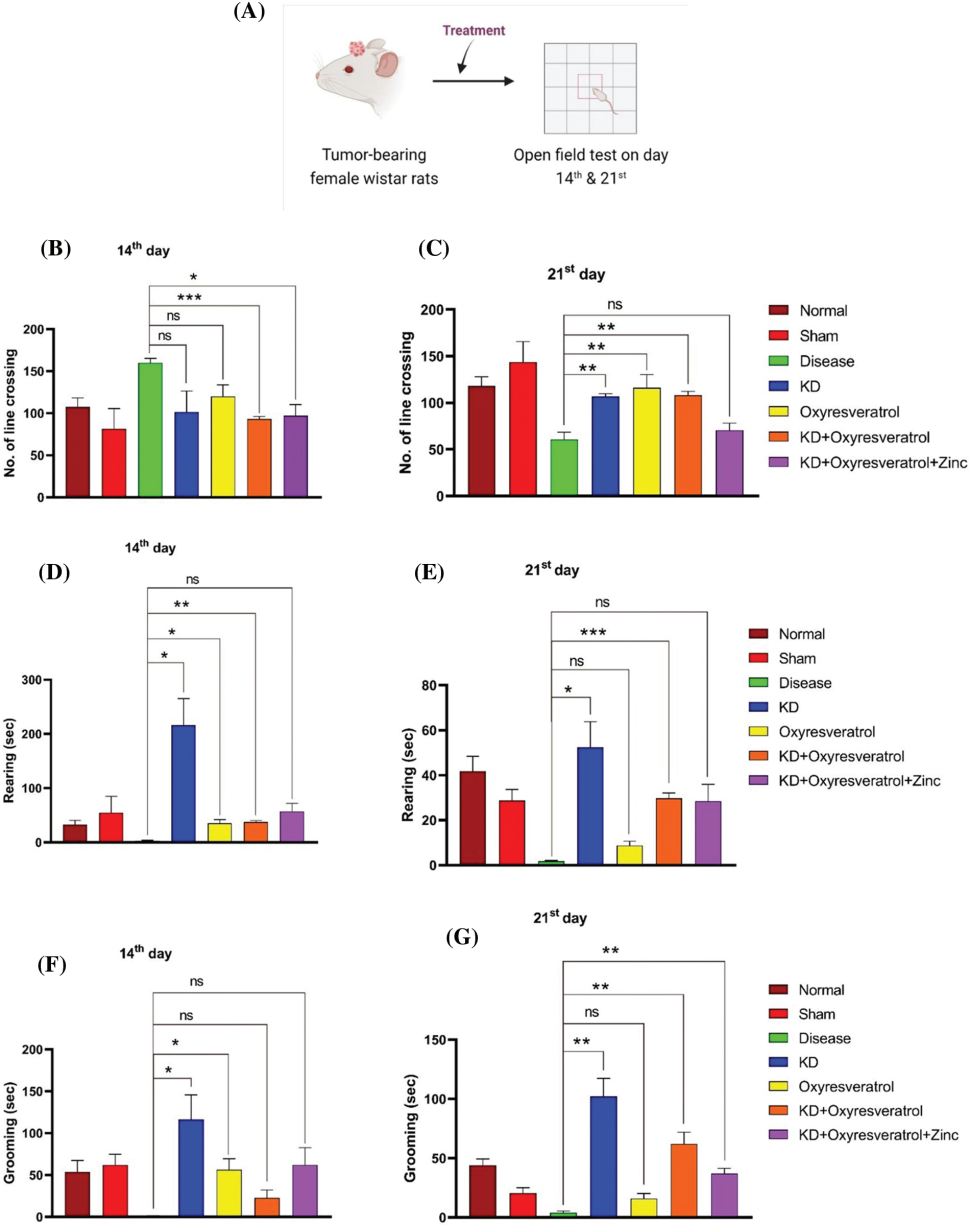
Assessment of locomotor activity in the animals using an open-field test (OFT). (A) Schematic representation showing the treatment of the animals and OFT performance on days 14 and 21. (B and C) Number of line crossings on days 14 and 21 (D and E) of rearing on days 14 and 21. (F and G) Estimation of grooming ability on the 14^th^ and 21^st^ days. The values are expressed as the means ± SEMs. Statistical analysis was performed using one-way ANOVA with Dunnett’s multiple comparisons test. **p < 0.01, ***p < 0.001 and *p < 0.05 compared to the disease control group.

The locomotor activity of the animals was estimated using an open field test (OFT) (Fig. 2A). Different parameters, such as time spent in the central square, time spent in the periphery squares, and the number of line crossings, rearings, and grooming events, were estimated. On the 14^th^ day, compared with the treatment groups, the disease control group showed an increased number of line crossings. There was a significant decrease in the number of line crossings in the groups treated with KD + oxyresveratrol and KD + oxyresveratrol + zinc (Fig. 2B). On the 21^st^ day of the study, the disease control group showed a decrease in the number of line crossings compared with the normal control group. There was a significant increase in the number of line crossings in the groups treated with KD, oxyresveratrol, and KD + oxyresveratrol compared with the disease control group (Fig. 2C). On day 14_,_ a decrease in rearing was observed in the disease control group compared with the normal control group. A significant increase in rearing behavior was observed in the groups treated with KD, oxyresveratrol, and KD + oxyresveratrol compared with the disease control group (Fig. 2D). On the 21^st^ day, a decrease in rearing was observed in the disease control group compared with the normal control group. A significant increase in rearing behavior was observed in the groups treated with KD and KD + oxyresveratrol compared with the disease control group (Fig. 2E).

**Figure 3:**
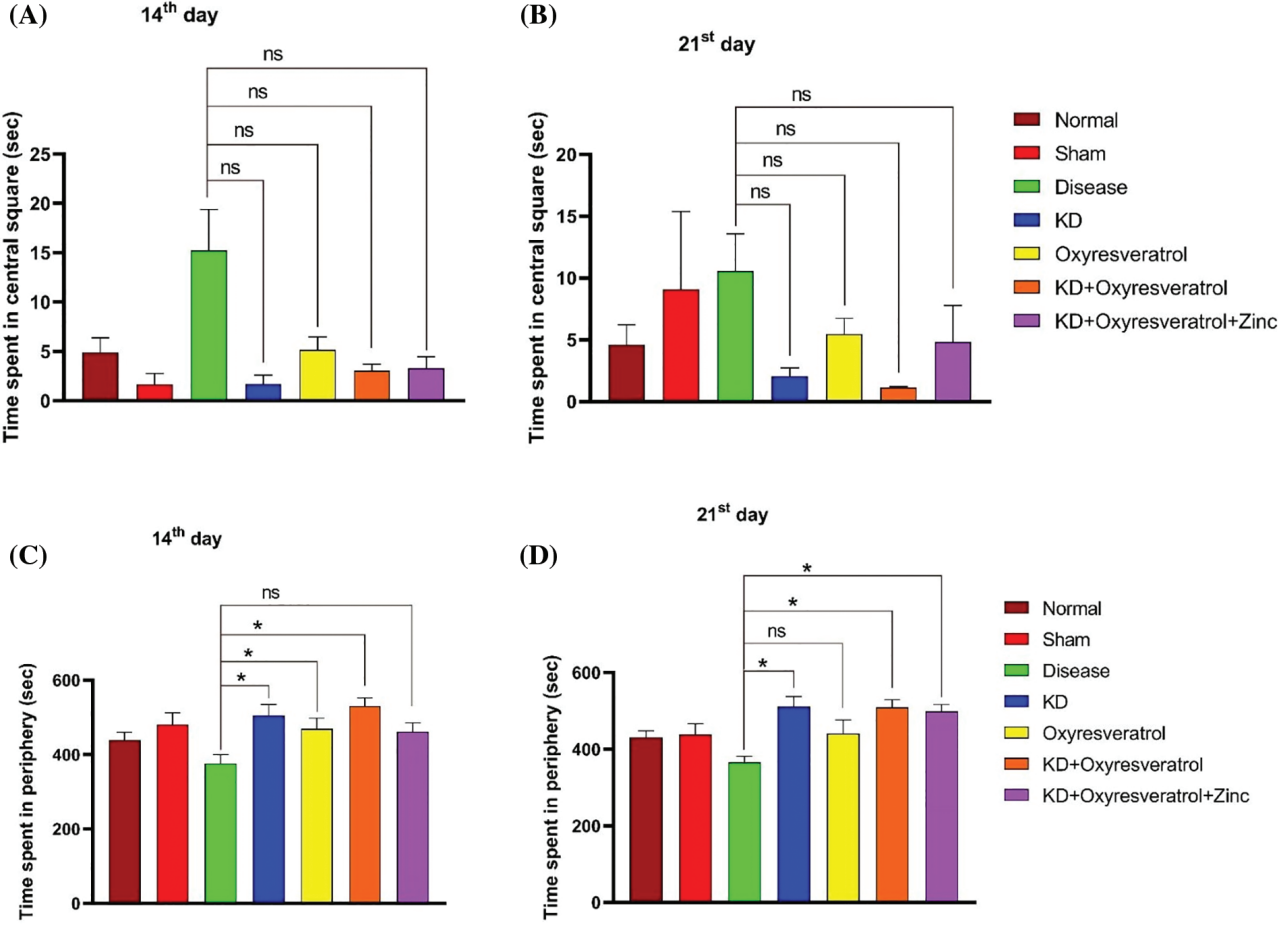
Estimation of time spent in the central square and periphery of animals using the OFT. (A and B) Time spent in the central square on days 14 and 21. (C and D) Time spent in the periphery on the 14^th^ and 21^st^ days. The values are expressed as the means ± SEMs. Statistical analysis was performed using one-way ANOVA with Dunnett’s multiple comparisons test. ns, not significant, *p < 0.05 compared to the disease control group.

A decrease in grooming behavior was observed in the disease control group compared with the normal control group. A significant increase in grooming behavior was observed in the KD and oxyresveratrol groups compared with the disease control group on the 14^th^ day (Fig. 2F). A decrease in grooming behavior was observed in the disease control group compared with the normal control group. A significant increase in grooming behavior was observed in the KD, KD + oxyresveratrol, and KD + oxyresveratrol + zinc groups compared to that in the disease control group on the 21^st^ day (Fig. 2G). There was more time spent in the central square in the disease control group than in the normal control group. A decrease in the time spent in the central square was observed in all the treatment groups compared with the disease control group on the 14^th^ day, but this decrease was not significant. The time spent in the central square was greater in the disease control group than in the normal control group (Fig. 3A). A decrease in the time spent in the central square was observed in all the treatment groups compared with the disease control group on the 21^st^ day (Fig. 3B), but this decrease was not significant. On day 14, a significant increase in the amount of time spent in the periphery was observed in the disease group compared with the KD, oxyresveratrol, and KD + oxyresveratrol groups (Fig. 3C). A significant difference was observed in the amount of time spent in the periphery between the disease group and the KD, KD + oxyresveratrol, and KD + oxyresveratrol + zinc groups on the 21^st^ day (Fig. 3D).

#### Rotarod

**Figure 4:**
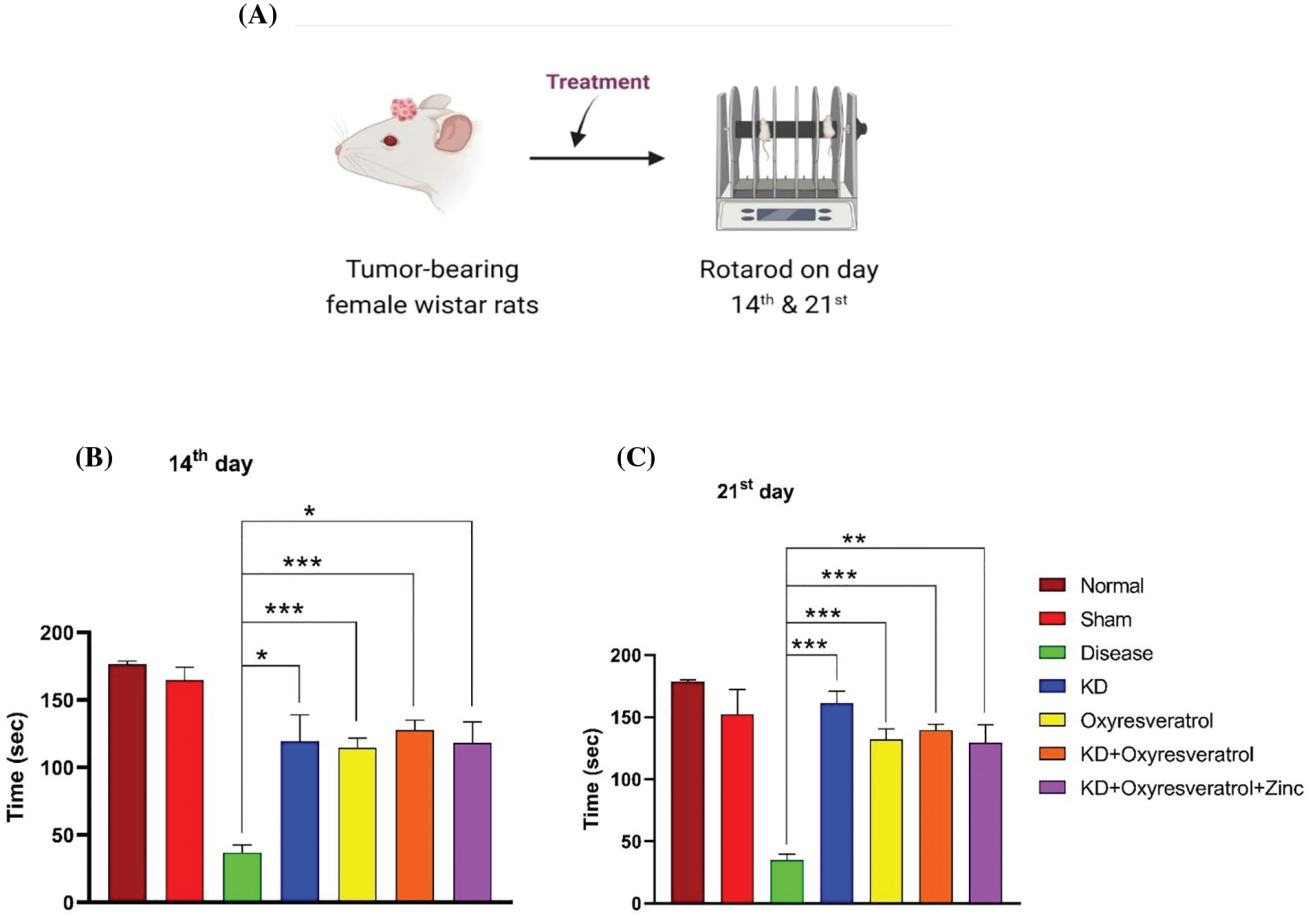
Evaluation of motor coordination in animals using the rotarod test. (A) Illustration showing the treatment of the animals and the performance of the rotarod test on days 14 and 21. (B and C) Time spent by animals on the rotarod on days 14 and 21. The values are expressed as the means ± SEMs. Statistical analysis was performed using one-way ANOVA with Dunnett’s multiple comparisons test. ***p < 0.001, **p < 0.01, *p < 0.05 compared to the disease control group.

To evaluate motor coordination, a rotarod test was performed (Fig. 4A). On day 14, the disease control group spent less time on the rotating rod than the normal group. Additionally, the KD, oxyresveratrol, KD + oxyresveratrol, and KD + oxyresveratrol + zinc treatment groups showed significant increases in time (Fig. 4B). The time spent on the rotating rod was significantly greater in all treatment group rats than in the disease control group (Fig. 4C).

### Effect of the ketogenic diet and its combination with oxyresveratrol and zinc on blood glucose and blood ketone levels in tumor-bearing animals

**Figure 5:**
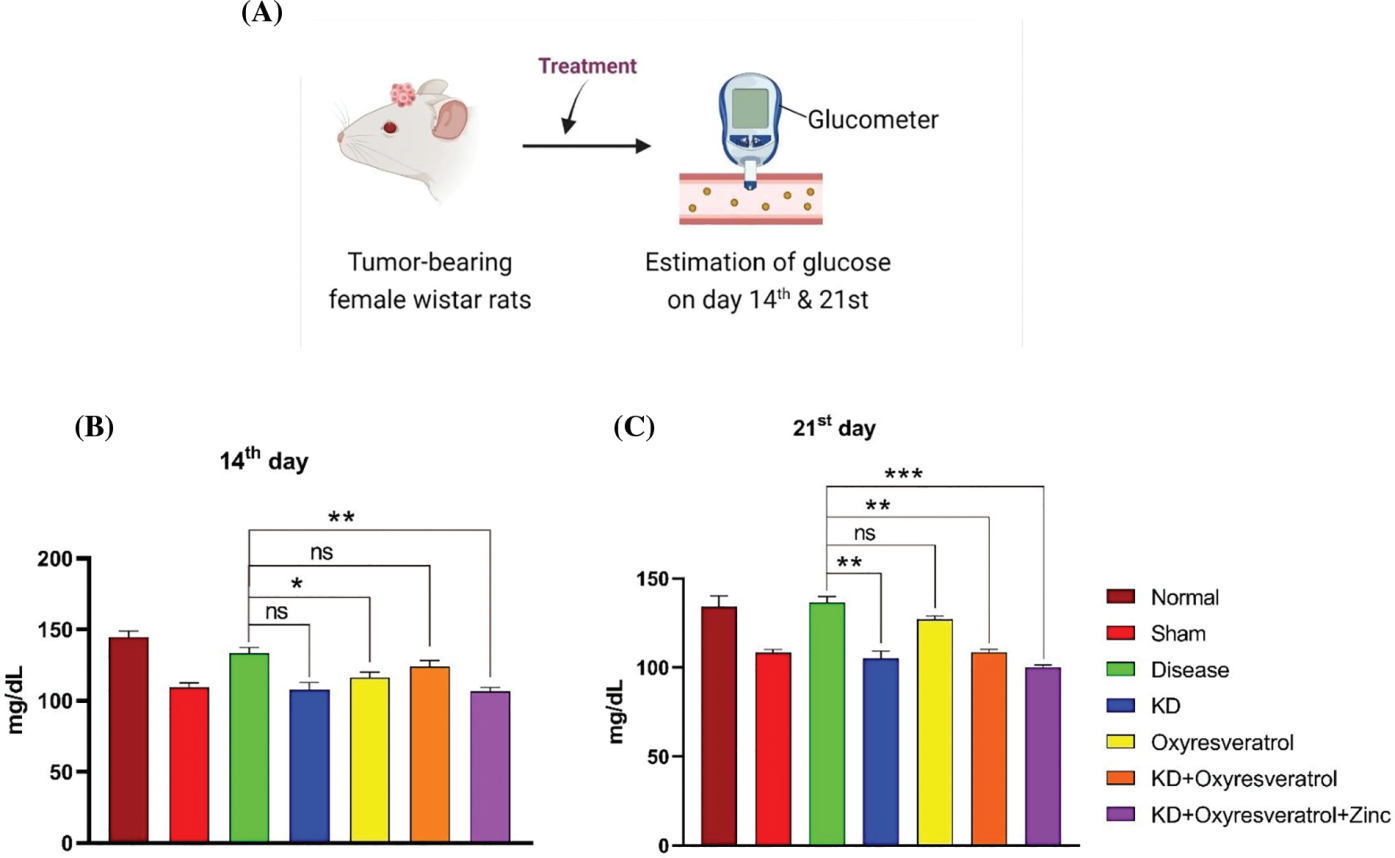
Estimation of blood glucose levels in animals. (A) Illustration showing the treatment of the animals and evaluation of glucose levels on days 14 and 21. (B and C) Estimated glucose levels (mg/dL) on days 14 and 21. The values are expressed as the means ± SEMs. Statistical analysis was performed using one-way ANOVA with Dunnett’s multiple comparisons test. ***p < 0.001, **p < 0.01 and *p < 0.05 compared to the disease control group.

**Figure 6:**
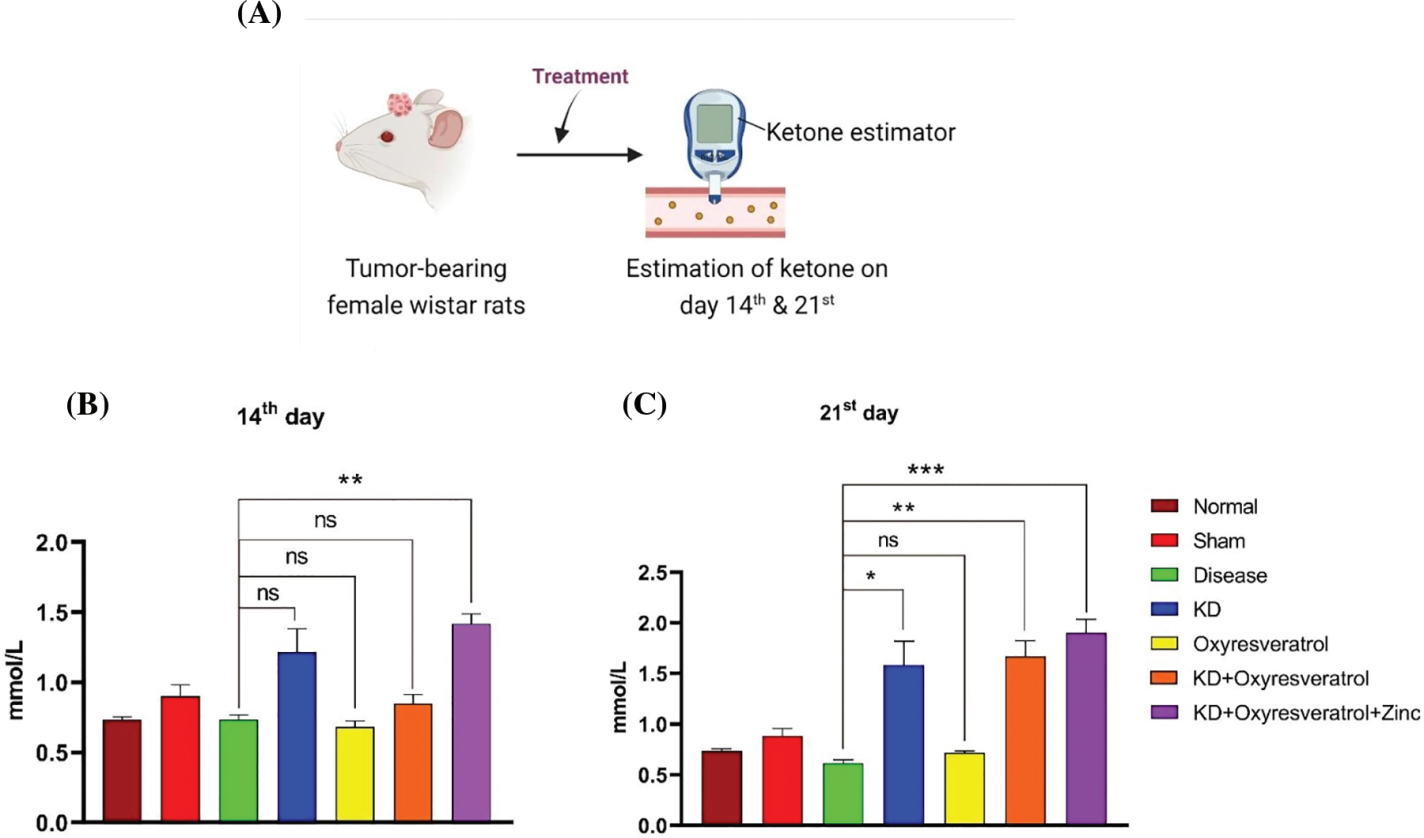
Estimation of ketone levels in animals. (A) Illustration showing the treatment of the animals and the evaluation of ketone levels on days 14 and 21. (B and C) Estimated ketone levels on days 14 and 21. The values are expressed as the means ± SEMs. Statistical analysis was performed using one-way ANOVA with Dunnett’s multiple comparisons test. ***p < 0.001, **p < 0.01 and *p < 0.05 compared to the disease control group.

Blood glucose and blood ketone levels were monitored weekly once (Fig. 5A). On the 14^th^ day of the study, the glucose level in the disease control group was slightly lower than that in the normal control group. Blood glucose levels were greater in the disease control group than in the other treatment groups. There was a significant decrease in blood glucose levels in the KD and KD + oxyresveratrol + zinc groups compared with those in the disease group (Fig. 5B). On the 21^st^ day of the study, a significant increase in the blood glucose level in the disease control group was observed compared with that in the normal control group, whereas a significant decrease in the blood glucose level was observed in the KD, KD + oxyresveratrol, and KD + oxyresveratrol + zinc groups compared with that in the disease group (Fig. 5C). Blood ketone levels were also monitored once a week (Fig. 6A). On the 14^th^ and 21^st^ days of the study, there was an increase in the blood ketone level in the groups treated with a KD compared with the normal and disease groups, which were fed a standard laboratory diet. Thus, there was a significant increase in the blood ketone levels in the KD, KD + oxyresveratrol, and KD + oxyresveratrol + zinc groups compared with those in the disease group (Fig. 6B,C).

### Zinc levels in the blood plasma of the animals

**Figure 7:**
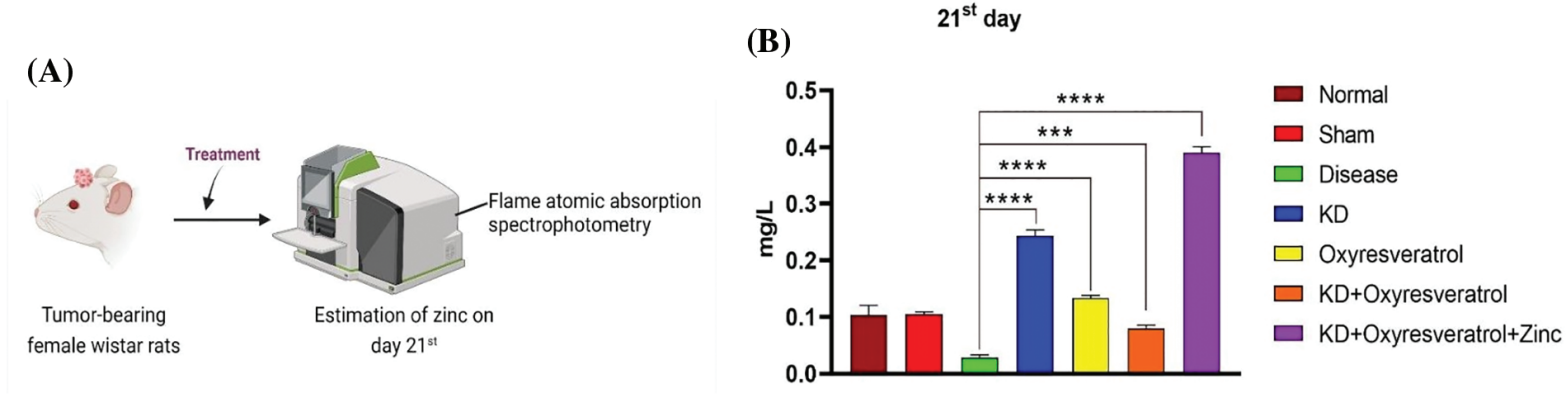
Evaluation of the zinc concentration in the animal blood plasma using flame atomic absorption spectrophotometry. (A) Schematic representation showing the treatment of the animals and the measurement of zinc on the 21^st^ day. (B) The level of zinc in the animal blood plasma (mg/L). The values are expressed as the means ± SEMs. Statistical analysis was performed using one-way ANOVA with Dunnett’s multiple comparisons test. ***p < 0.001, ****p < 0.0001 compared to the disease control group.

To determine the level of zinc in the blood plasma of the animals, flame atomic absorption spectroscopy was performed (Fig. 7A). On the 21^st^ day, a significant decrease in the zinc level in the disease control group was observed compared with that in the normal control group. Compared with the disease control group, the KD + oxyresveratrol + zinc group exhibited a significant increase in the plasma zinc level (Fig. 7B).

### Effect of the ketogenic diet and its combination with oxyresveratrol and zinc on tumor volume

**Figure 8:**
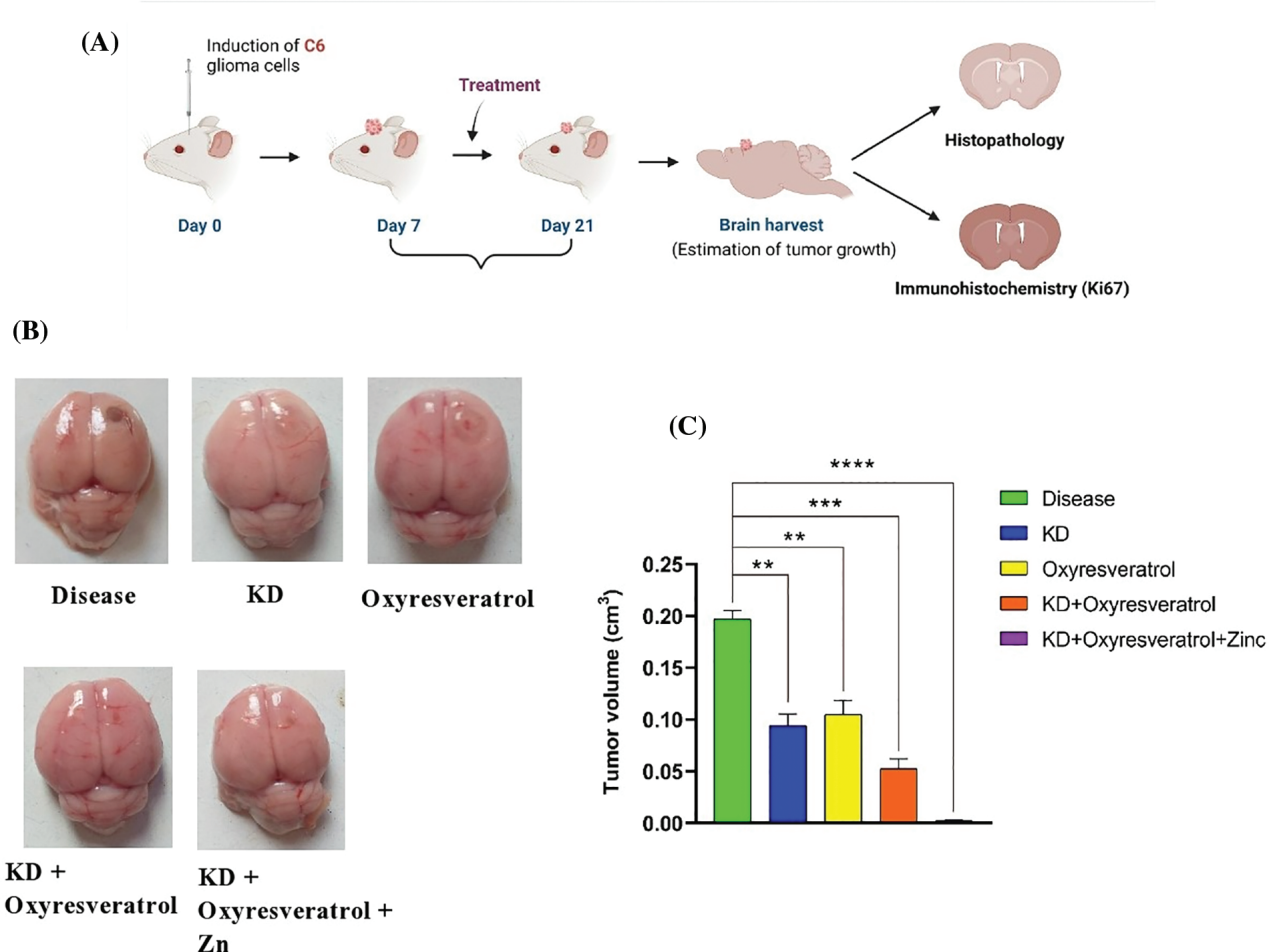
Induction of C6 glioma cells in the animals and evaluation of tumor growth. (A) Schematic representation showing tumor cell induction in female Wistar rats and estimation of tumor progression on days 14 and 21. (B) Brains isolated from the animals in the treated groups showing tumor progression and inhibited tumor growth. (C) Estimation of the tumor volume (cm^3^) on the 21^st^ day. The values are expressed as the means ± SEMs. Statistical analysis was performed using one-way ANOVA with Dunnett’s multiple comparisons test. **p < 0.01, ***p < 0.001 and ****p < 0.0001 compared to the disease control group.

After 21 days of the study, all the animals were humanely euthanized, and their brains were removed for further analysis (Fig. 8A). Compared with the disease control group, the disease control group showed significant tumor growth. There was a significant decrease in the tumor volume in the groups treated with KD, oxyresveratrol, KD + oxyresveratrol, and KD + oxyresveratrol + zinc compared with the disease control group. However, compared with the other treatment groups, the KD + oxyresveratrol + zinc group exhibited massive tumor suppression (Fig. 8B,C).

### Tissue examination and immunohistochemical analysis

**Figure 9:**
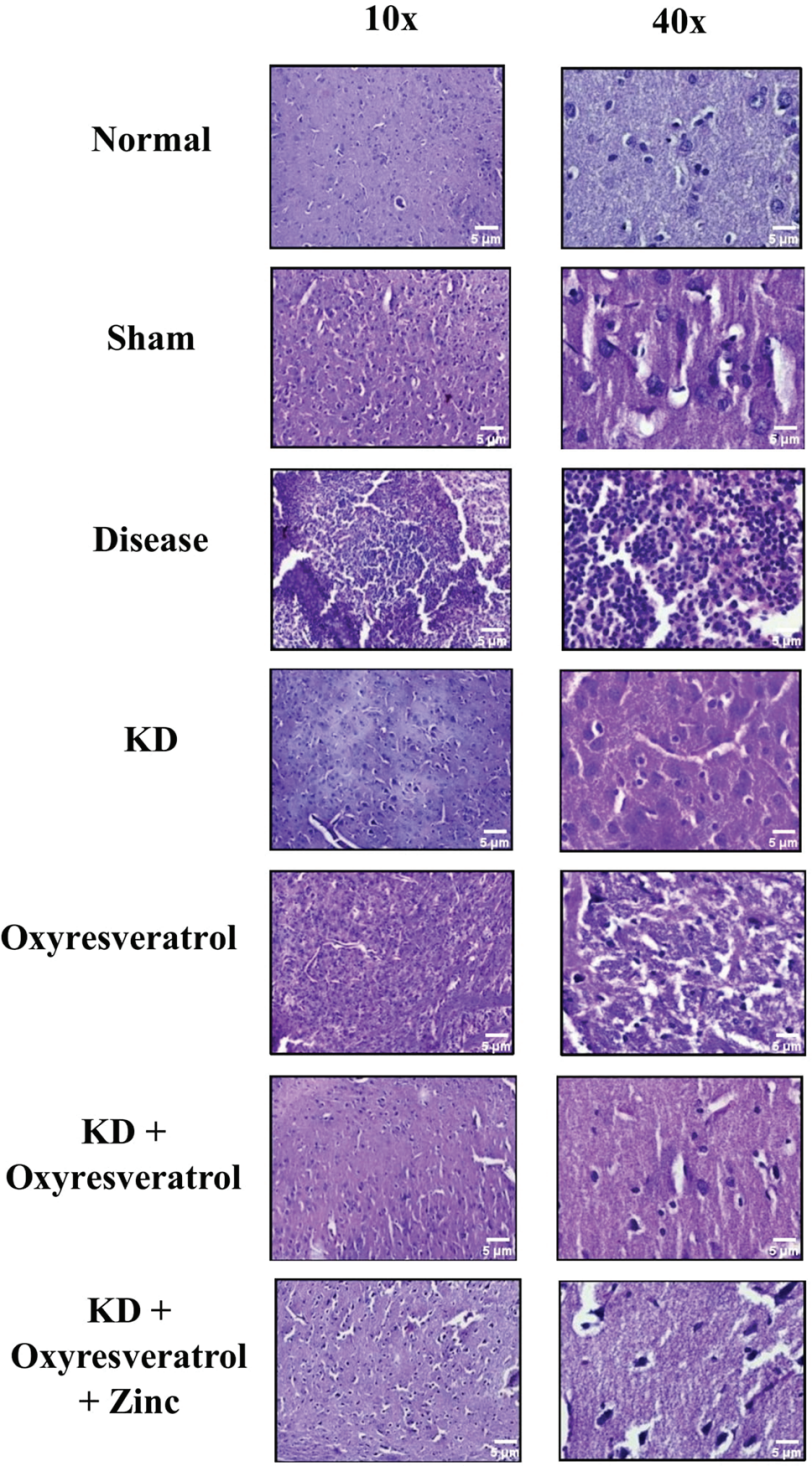
Histopathological analysis. Histopathological estimation revealed changes in the tumor burden following treatment, which were visualized under a confocal microscope at 10× and 40×. The thickness of the tissue analyzed was 5 µm.

Tissue examination was performed using hematoxylin and eosin (H&E) staining. Histopathology studies revealed that the normal and sham groups exhibited normal and functional cell structures. In disease control, a large number of tumor cells were found and clearly showed the induction of tumors in the animals. The intensity of tumor growth in the groups treated with the KD and the combination of the KD with oxyresveratrol and zinc was very low compared with that in the disease control group. Moreover, the histopathological results of the KD group, KD + oxyresveratrol group, and KD + oxyresveratrol + zinc group showed that the KD + oxyresveratrol + zinc treatment was very effective at reducing tumor cell growth, and the tumor burden was less than that of the disease control group (Fig. 9).

**Figure 10:**
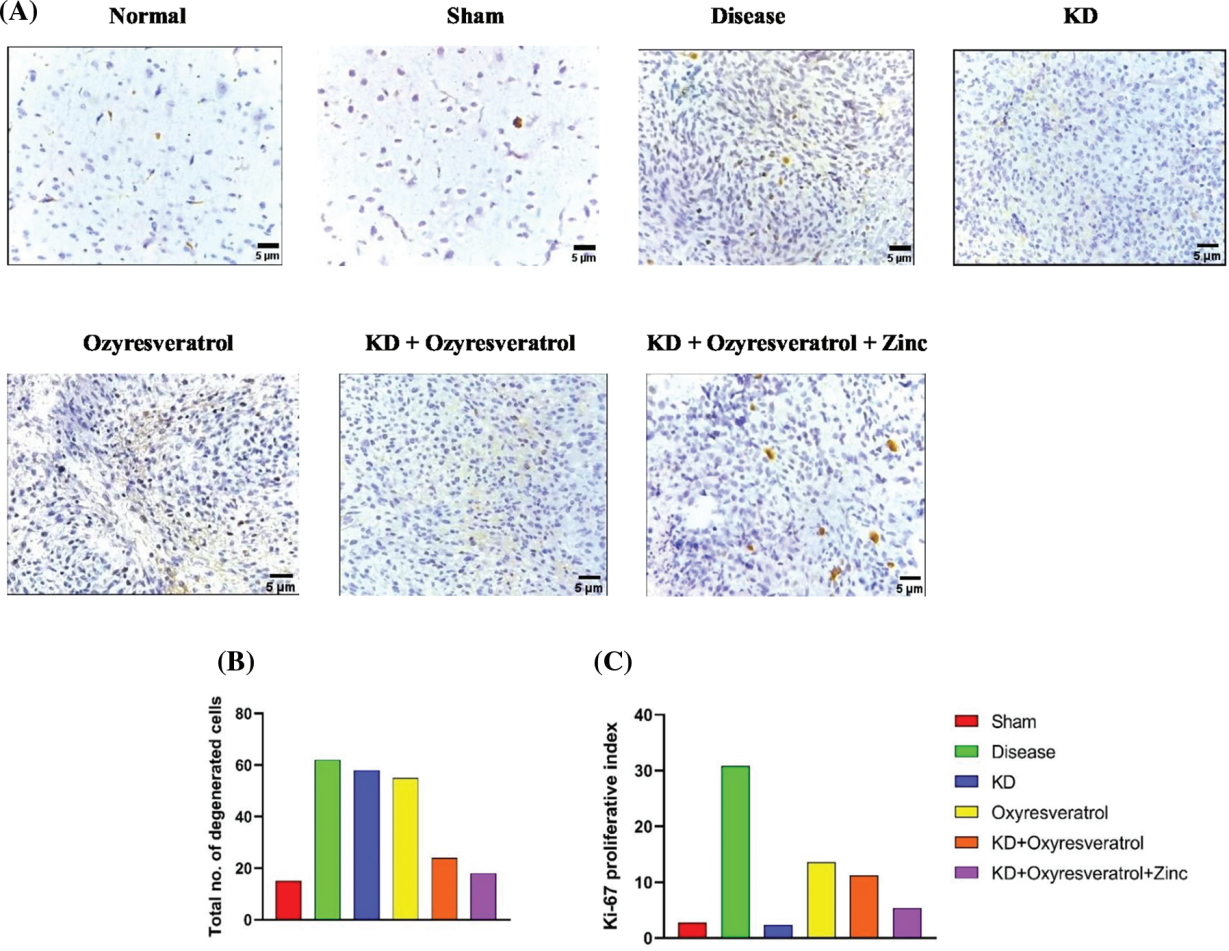
Immunohistochemistry analysis. (A) Immunohistochemical analysis showing changes in the tumor burden, (B) the total number of degenerated cells, and (C) the Ki-67 proliferation marker following treatment; the results were visualized under a confocal microscope at 400×. The thickness of the tissue analyzed was 5 µm.

IHC analysis revealed that the number of degenerated cells and the Ki-67 proliferative index were greater in the disease control group than in the sham and normal control groups. Fewer degenerated cells were observed in the group treated with KD + oxyresveratrol + zinc (Fig. 10A,B). A smaller number of Ki-67 markers were detected in the KD group than in the other treatment groups (Fig. 10A,C).

## Discussion

GBM is the most aggressive CNS tumor characterized by high invasiveness, elevated vascularity, and a propensity to disperse throughout the brain parenchyma, resulting in short overall survival (OS) and progression-free survival (PFS) rates. The availability of the current treatment regimen has not led to a permanent cure for the disease [54]. Many patients have either developed resistance to the current therapy or have a high chance of disease reoccurrence [55,56]. As discussed before, many studies have highlighted that GBM cells are highly dependent on glucose for metabolism, which is the energy required for the differentiation and proliferation of cancer cells. Henceforth, new approaches are being developed to alter the metabolic requirements of cancer cells. KD alone and in combination with other chemotherapeutic agents has been shown to have anticancer effects and thus to inhibit the growth of tumors [57–59].

This study hypothesized that KD and zinc-modulated Oxy-resveratrol have anticancer effects by reversing the metabolic and epigenetic abnormalities present in GBM, repressing tumor growth, and combating tumor progression. Furthermore, these effects improve memory and motor function affected by tumor growth. The present study was performed to investigate the anticancer effect of a ketogenic diet, as well as the combined effect of a KD and zinc, and modulated oxyresveratrol against C6-induced glioma in female Wistar rats. On day 0, stereotaxic surgery was performed to induce GBM. On the 6^th^ day after surgery, a normal diet was given to all the groups. From the 7^th^ day onwards until the 21^st^ day, the KD group was divided into 3 groups: the KD group, the KD + oxyresveratrol group, and the KD + oxyresveratrol + zinc group. Oxyresveratrol was administered to the KD + oxyresveratrol and Oxyresveratrol groups, and zinc was administered to the KD + oxyresveratrol + zinc group from the 7^th^ day to the 21^st^ day. The Morris water maze (MWM) was used to evaluate memory function, and the rotarod test and OFT were performed to assess motor coordination in all the groups on days 14 and 21.

Escape latency and path efficiency were evaluated in the MWM test. After 21 days of treatment, a significant difference in escape latency was observed between the disease group and the KD, oxyresveratrol, KD + oxyresveratrol, and KD + oxyresveratrol + zinc groups. The escape latency of the disease group was greater than that of the other treatment groups. This shows that the KD was effective in reducing the escape latency compared to that of the disease group; thus, the memory of the KD-treated group was intact compared with that of the disease group. The path efficiency of the disease group decreased compared with that of the other groups treated with KD alone or in combination with oxyresveratrol and zinc. Thus, a significant difference was observed in path efficiency between the disease group and the KD and KD + xyresveratrol groups. Hence, from this result, it could be inferred that the memory of the disease group was affected, and the treatment groups showed improvement in memory function.

The motor coordination of the disease group was affected compared with that of the normal and sham groups. This shows that GBM impairs the motor function of animals. When the rotarod data from the 14^th^ and 21^st^ days were compared, on day 21, the KD group exhibited improved motor function. The OFT was also performed to evaluate the motor function of the Wistar rats, and the motor function of the disease group was impaired compared with that of the other treatment groups. Initially, the number of line crossings was lower in all treatment groups, but by the end of the study, the KD, oxyresveratrol, and KD + oxyresveratrol groups showed a significant increase in the number of line crossings compared with the disease group. All the treatment groups demonstrated increased rearing activity in comparison to the disease group. A significant increase in grooming was observed in the KD-, KD + oxyresveratrol-, and KD + oxyresveratrol + zinc-treated groups compared with the disease group. Animals in the disease group spent more time in the central square, which could be due to the affected locomotor movement, whereas the groups treated with KD, KD + oxyresveratrol, and KD + oxyresveratrol + zinc spent most of their time in the periphery square.

Zinc plays a pivotal role in the epigenetic modulation of GBM. Zinc plays an important role in reducing tumor growth and proliferation. In our study, we observed that the level of zinc decreased in the disease group. The tumor volume was significantly lower in the KD + oxyresveratrol + zinc-treated group than in the disease group or the other treatment groups. Cancer cells exhibit altered metabolism compared with that of normal cells. Hence, there is increased utilization of glucose by cancer cells for growth and proliferation compared with that of normal cells [60]. Ketone bodies can be used as a source of energy by normal cells, whereas tumors cannot utilize ketone bodies as an alternative source of energy when the supply of glucose is limited. This is because tumor cells lack ketone-metabolizing enzymes [61]. In this study, there was a significant decrease in the glucose level in all the groups treated with the KD compared with the disease group. The ketone body levels were estimated, and it was found that the disease group had decreased ketone levels, whereas the ketone levels of the groups treated with the KD for 21 days were significantly greater than those of the normal group. From the histopathological results, it is clear that in disease control, there is an increased tumor burden. The density, as well as the intensity of tumor cell growth, was found to be very low in the KD + oxyresveratrol + zinc group compared to that in the disease control group. An immunohistochemical analysis also confirmed that in the disease control group, there were highly degenerated cells and a proliferative marker, Ki-67, in the tumor cells, and it was also shown that the KD group had a lower tumor burden and Ki-67 index than the other treatment groups. Hence, the combination of a KD with oxyresveratrol and zinc was effective in reducing GBM in female Wistar rats. However, there are certain limitations associated with this study which can be explored in the future including monitoring the absorption of the KD, visualization of the tumor growth and inhibition using *in vivo* bioluminescence imaging technique, protein expression analysis for epigenetic markers using western blot, and flow cytometry analysis to predict the cell cycle inhibition.

## Conclusion

In conclusion, compared with the disease control group and the other treatment groups, the KD + oxyresveratrol + zinc combination group exhibited decreased tumor growth. Overall memory function and motor coordination were also greater in the KD, oxyresveratrol, KD + oxyresveratrol, and KD + oxyresveratrol + zinc groups than in the disease control group. In addition, the KD group had lower Ki-67 levels than the other treatment groups. Hence, our study proved that KD has anticancer efficacy, and that zinc is a potential epigenetic modulator of Oxyresveratrol that promotes the inhibition of GBM cells and prevents tumor growth.

## Data Availability

Data will be made available on request.

## References

[1] Masoudi, M. S., Mehrabian, E., Mirzaei, H. (2018). MiR-21: A key player in glioblastoma pathogenesis. Journal of Cellular Biochemistry*,* 119 *(*2*),* 1285–1290. 10.1002/jcb.26300; 28727188

[2] Schwartz, K. A., Noel, M., Nikolai, M., Chang, H. T. (2018). Investigating the ketogenic diet as treatment for primary aggressive brain cancer: Challenges and lessons learned. Frontiers in Nutrition*,* 5*,* 11. 10.3389/fnut.2018.00011; 29536011 PMC5834833

[3] Fang, X. G., Huang, Z., Zhou, W. C., Wu, Q. L., Sloan, A. E. et al. (2014). The zinc finger transcription factor ZFX is required for maintaining the tumorigenic potential of glioblastoma stem cells. Stem Cells*,* 32 *(*8*),* 2033–2047. 10.1002/stem.1730; 24831540 PMC4349564

[4] Ostrom, Q. T., Cioffi, G., Waite, K., Kruchko, C., Barnholtz-Sloan, J. S. (2021). CBTRUS statistical report: Primary brain and other central nervous system tumors diagnosed in the united states in 2014–2018. Neuro Oncology*,* 23 *(*12 Suppl 2*),* iii1–iii105. 10.1093/neuonc/noab200; 34608945 PMC8491279

[5] Dasgupta, A., Gupta, T., Jalali, R. (2016). Indian data on central nervous tumors: A summary of published work. South Asian Journal of Cancer*,* 5 *(*3*),* 146–147. 10.4103/2278-330X.187589; 27606302 PMC4991137

[6] Pathak, N., Cheruku, S. P., Rao, V., Vibhavariet, R. J. A., Sumalatha, S. et al. (2020). Dehydrozingerone protects temozolomide-induced cognitive impairment in normal and C6 glioma rats besides enhancing its anticancer potential. 3 Biotech*,* 10*,* 438; 32995109 10.1007/s13205-020-02427-7 PMC7498526

[7] Buyandelger, B., Bar, E. E., Hung, K. S., Chen, R. M., Chiang, Y. H. et al. (2020). The histone deacetylase inhibitor MPT0B291 suppresses glioma growth *in vitro* and *in vivo* partially through acetylation of p53. International Journal of Biological Sciences*,* 16 *(*6*),* 3184–3199; 33162824 10.7150/ijbs.45505 PMC7645997

[8] Lee, P., Murphy, B., Miller, R., Menon, V., Banik, N. L. et al. (2015). Mechanisms and clinical significance of histone deacetylase inhibitors: Epigenetic glioblastoma therapy. Anticancer Research*,* 35 *(*2*),* 615–625; 25667438 PMC6052863

[9] Le, T. N. T., Lim, H., Hamilton, A. M., Parkins, K. M., Chen, Y. et al. (2018). Characterization of an orthotopic rat model of glioblastoma using multiparametric magnetic resonance imaging and bioluminescence imaging. Tomography*,* 4 *(*2*),* 55–65; 30206545 10.18383/j.tom.2018.00012 PMC6127346

[10] Lussier, D. M., Woolf, E. C., Johnson, J. L., Brooks, K. S., Blattman, J. S. et al. (2016). Enhanced immunity in a mouse model of malignant glioma is mediated by a therapeutic ketogenic diet. BMC Cancer*,* 16*,* 310; 27178315 10.1186/s12885-016-2337-7 PMC4866042

[11] Wu, Z., Nakamura, M., Krauss, J. K., Schwabe, K., John, N. (2018). An intracranial rat glioma model for tumor resection and local treatment. Journal of Neuroscience Methods*,* 299*,* 1–7; 29425709 10.1016/j.jneumeth.2018.02.002

[12] Martin-McGill, K. J., Marson, A. G., Tudur Smith, C., Young, B., Mills, S. J. et al. (2020). Ketogenic diets as an adjuvant therapy for glioblastoma (KEATING): A randomized, mixed methods, feasibility study. Journal of Neurooncology*,* 147 *(*1*),* 213–227; 32036576 10.1007/s11060-020-03417-8 PMC7076054

[13] Varshneya, K., Carico, C., Ortega, A., Patil, C. G. (2015). The efficacy of ketogenic diet and associated hypoglycemia as an adjuvant therapy for high-grade gliomas: A review of the literature. Cureus*,* 7 *(*2*),* e251; 26180675 10.7759/cureus.251 PMC4494562

[14] Young, R. M., Jamshidi, A., Davis, G., Sherman, J. H. (2015). Current trends in the surgical management and treatment of adult glioblastoma. Annals of Translational Medicine*,* 3 *(*9*),* 121; 26207249 10.3978/j.issn.2305-5839.2015.05.10 PMC4481356

[15] Pearson, J. R. D., Regad, T. (2017). Targeting cellular pathways in glioblastoma multiforme. Signal Transduction and Targeted Therapy*,* 2*,* 17040; 29263927 10.1038/sigtrans.2017.40 PMC5661637

[16] Zhao, T., Li, C., Ge, H., Lin, Y., Kang, D. (2022). Research progress in glioblastoma vaccine-based tumor therapy. Chinese Neurosurgical Journal*,* 8 *(*2*),* 1–5 (In Chinese). 35045874 10.1186/s41016-021-00269-7 PMC8766628

[17] Festuccia, C., Biordi, A. L., Tombolini, V., Hara, A., Bailey, D. (2020). Targeted molecular therapy in glioblastoma. Journal of Oncology*,* 2020*,* 5104876. 10.1155/2020/5104876; 32411237 PMC7201507

[18] Tseng, Y. Y., Huang, Y. C., Yang, T. C., Yang, S. T., Liu, S. C. et al. (2016). Concurrent chemotherapy of malignant glioma in rats by using multidrug-loaded biodegradable nanofibrous membranes. Scientific Reports*,* 6*,* 30630. 10.1038/srep30630; 27471070 PMC4965810

[19] Maroon, J., Seyfried, T., Donohue, J., Bost, J. (2015). The role of metabolic therapy in treating glioblastoma multiforme. Surgical Neurology International*,* 6, 61; 25949849 10.4103/2152-7806.155259 PMC4405891

[20] Woolf, E. C., Syed, N., Scheck, A. C. (2016). Tumor metabolism, the ketogenic diet and β-hydroxybutyrate: Novel approaches to adjuvant brain tumor therapy. Frontiers in Molecular Neuroscience*,* 9*,* 122. 10.3389/fnmol.2016.00122; 27899882 PMC5110522

[21] Maeyama, M., Tanaka, K., Nishihara, M., Irino, Y., Shinohar, M. et al. (2021). Metabolic changes and antitumour effects of a ketogenic diet combined with antiangiogenic therapy in a glioblastoma mouse model. Scientific Reports*,* 11*,* 79; 33420169 10.1038/s41598-020-79465-x PMC7794443

[22] Mukherjee, P., Augur, Z. M., Li, M., Hill, C., Greenwood, B. et al. (2019). Therapeutic benefit of combining calorie-restricted ketogenic diet and glutamine targeting in late-stage experimental glioblastoma. Communications Biology*,* 2*,* 200. 10.1038/s42003-019-0455-x; 31149644 PMC6541653

[23] Tran, Q., Lee, H., Kim, C., Kong, G., Gong, N. et al. (2020). Revisiting the Warburg effect: Diet-based strategies for cancer prevention. Biomed Research International. 10.1155/2020/8105735; 32802877 PMC7426758

[24] Weber, D. D., Kofler, B. (2018). Ketogenic diet in cancer therapy *Aging*, 10 *(*2*),* 164–165. 10.18632/aging.101382; 29443693 PMC5842847

[25] Arora, N., Mehta, T. R. (2020). Role of the ketogenic diet in acute neurological diseases. Clinical Neurology and Neurosurgery*,* 192, 105727. 10.1016/j.clineuro.2020.105727; 32087500

[26] Nguyen, T. T. T., Zhang, Y., Shang, E., Shu, C., Torrini, C. et al. (2020). HDAC inhibitors elicit metabolic reprogramming by targeting superenhancers in glioblastoma models. Journal of Clinical Investigation*,* 130 *(*7*),* 3699–3716. 10.1172/JCI129049; 32315286 PMC7324177

[27] Pant, K., Richard, S., Peixoto, E., Gradilone, S. A. (2020). Role of glucose metabolism reprogramming in the pathogenesis of cholangiocarcinoma. Frontiers in Medicine. 7*,* 113. 10.3389/fmed.2020.00113; 32318579 PMC7146077

[28] Jensen, N. J., Wodschow, H. Z., Nilsson, M., Rungby, J. (2020). Effects of ketone bodies on brain metabolism and function in neurodegenerative diseases. International Journal of Molecular Sciences*,* 21 *(*22*),* 8767. 10.3390/ijms21228767; 33233502 PMC7699472

[29] Sperry, J., Condro, M. C., Guo, L., Braas, D., Harris, N. et al. (2020). Glioblastoma utilizes fatty acids and ketone bodies for growth allowing progression during ketogenic diet therapy. iScience*,* 23 *(*9*),* 101453. 10.1016/j.isci.2020.101453; 32861192 PMC7471621

[30] Santos, J. G., da Cruz, W. M. S., Schönthal, A. H., Salazar, M. D., Fontes, C. A. P. et al. (2018). Efficacy of a ketogenic diet with concomitant intranasal perillyl alcohol as a novel strategy for the therapy of recurrent glioblastoma. Oncology Letters*,* 15*,* 1263–1270. 29391903 10.3892/ol.2017.7362 PMC5769394

[31] Ji, C. C., Hu, Y. Y., Cheng, G., Liang, L., Gao, B. et al. (2020). A KD attenuates the proliferation and stemness of glioma stem-like cells by altering metabolism, resulting in increased ROS production. International Journal of Oncology*,* 56*,* 606–617; 31894296 10.3892/ijo.2019.4942

[32] Martuscello, R. T., Vedam-Mai, V., McCarthy, D. J., Schmoll, M. E., Jundi, M. A. et al. (2016). A supplemented high-fat low-carbohydrate diet for the treatment of glioblastoma. Clinical Cancer Research*,* 22 *(*10*),* 2482–2495. 10.1158/1078-0432.CCR-15-0916; 26631612

[33] Aminzadeh-Gohari, S., Feichtinger, R. G., Vidali, S., Locker, F., Rutherford, T. et al. (2017). A ketogenic diet supplemented with medium-chain triglycerides enhances the antitumour and antiangiogenic efficacy of chemotherapy on neuroblastoma xenografts in a CD1-nu mouse model. Oncotarget*,* 8 *(*39*),* 64728–64744. 10.18632/oncotarget.20041; 29029389 PMC5630289

[34] Martin-McGill, K. J., Marson, A. G., Tudur Smith, C., Jenkinson, M. D. (2018). The modified ketogenic diet in adults with glioblastoma: An evaluation of feasibility and deliverability within the national health service. Nutrition and Cancer*,* 70 *(*4*),* 643–649. 10.1080/01635581.2018.1460677; 29668317

[35] Woolf, E. C., Curley, K. L., Liu, Q., Turner, G. H., Charlton, J. A. et al. (2015). A KD alters the hypoxic response and affects the expression of proteins associated with angiogenesis, invasive potential and vascular permeability in a mouse glioma model. PLoS One*,* 10*,* 1–18. 10.1371/journal.pone.0130357 PMC4470583 26083629

[36] Likhitwitayawuid, K. (2021). Oxyresveratrol: Sources, productions, biological activities, pharmacokinetics, and delivery systems. Molecules*,* 26 *(*14*),* 4212. 10.3390/molecules26144212; 34299485 PMC8307110

[37] Öztürk, Y., Günaydın, C., Yalçın, F., Nazıroğlu, M., Braidy, N. (2019). Resveratrol enhances the apoptotic and oxidant effects of paclitaxel through TRPM2 channel activation in DBTRG glioblastoma cells. Oxidative Medicine and Cellular Longevity*,* *2019*, 1–13. 10.1155/2019/4619865 PMC6431513 30984336

[38] Zhai, K., Siddiqui, M., Abdellatif, B., Liskova, A., Kubatka, P. et al. (2021). Natural compounds in glioblastoma therapy: Preclinical insights, mechanistic pathways, and outlook. Cancers*,* 13 *(*10*),* 1–36. 10.3390/cancers13102317; 34065960 PMC8150927

[39] Yuan, Y., Xue, X., Guo, R. B., Sun, X., Hu, L. et al. (2012). Resveratrol enhances the antitumour effects of temozolomide in glioblastoma via ROS-dependent AMPK-TSC-mTOR signalling pathway. CNS Neuroscience & Therapeutics*,* 18 *(*7*),* 536–546. 10.1111/j.1755-5949.2012.00319.x; 22530672 PMC6493617

[40] Lorenz, P., Roychowdhury, S., Engelmann, M., Wolf, G., Horn, T. F. W. (2003). Oxyresveratrol and resveratrol are potent antioxidants and free radical scavengers: Effect on nitrosative and oxidative stress derived from microglia. Nitric Oxide*,* 9 *(*2*),* 64–76. 10.1016/j.niox.2003.09.005; 14623172

[41] Su, Y., Sun, C., Chen, Y., Liu, S., Jing, N. et al. (2019). Oxyresveratrol intercepted toxic trans-crotonaldehyde in mitochondria, contributing to anticancer effects. IUBMB Life*,* 71 *(*7*),* 1014–1020. 10.1002/iub.2051. 31012998

[42] Yang, Y., Zhang, G., Li, C., Wang, S., Zhu, M. et al. (2019). Metabolic profile and structure-activity relationship of resveratrol and its analogues in human bladder cancer cells. Cancer Management and Research*,* 11*,* 4631–4642. 10.2147/CMAR 31191024 PMC6535101

[43] Balaji, E. V., Kumar, N., Satarker, S., Nampoothiri, M. (2020). Zinc as a plausible epigenetic modulator of glioblastoma multiforme. European Journal of Pharmacology*,* 887*,* 173549. 10.1016/j.ejphar.2020.173549; 32926916

[44] Choi, S., Cui, C., Luo, Y., Kim, S. H., Ko, J. K. et al. (2018). Selective inhibitory effects of zinc on cell proliferation in esophageal squamous cell carcinoma through Orai1. FASEB Journal*,* 32 *(*1*),* 404–416. 10.1096/fj.201700227RRR; 28928244 PMC6207365

[45] Bafaro, E., Liu, Y., Xu, Y., Dempski, R. E. (2017). The emerging role of zinc transporters in cellular homeostasis and cancer. Signal Transduction and Targeted Therapy*,* 2*,* 17029. 10.1038/sigtrans.2017.29; 29218234 PMC5661630

[46] Baraka, A. M., Hassab El Nabi, W., el Ghotni, S., (2012). Investigating the role of zinc in a rat model of epilepsy. CNS Neuroscience & Therapeutics*,* 18 *(*4*),* 327–333. 10.1111/j.1755-5949.2011.00252.x; 22070383 PMC6493370

[47] Skrajnowska, D., Bobrowska-Korczak, B. (2019). Role of zinc in immune system and anticancer defense mechanisms. Nutrients*,* 11 *(*10*),* 2273; 31546724 10.3390/nu11102273 PMC6835436

[48] Dimitropoulou, P., Nayee, S., Liu, J. F., Demetriou, L., Tongeren, M. V. et al. (2008). Dietary zinc intake and brain cancer in adults: A case‒control study. British Journal of Nutrition*,* 99 *(*3*),* 667–673. 10.1017/S0007114507831692; 17908366

[49] Skrajnowska, D., Korczak, B. B., Tokarz, A., Kazimierczuk, A., Klepacz, M. et al. (2015). The effect of zinc and phytoestrogen supplementation on the changes in mineral content of the femur of rats with chemically induced mammary carcinogenesis. Journal of Trace Elements in Medicine and Biology*,* 32 *(*12*),* 79–85. 10.1016/j.jtemb.2015.06.004; 26302916

[50] Sun, L., Gu, L., Wang, S., Yuan, J., Yang, H. et al. (2012). N-acetylcysteine protects against apoptosis through modulation of group i metabotropic glutamate receptor activity. PLoS One*,* 7 *(*3*),* 1–11. 10.1371/journal.pone.0032503; 22442667 PMC3307713

[51] Li, J., Liu, M., Gao, J., Jiang, Y., Wu, L. et al. (2020). AVNP2 protects against cognitive impairments induced by C6 glioma by suppressing tumour-associated inflammation in rats. Brain Behavior and Immunity*,* 87 *(*4*),* 645–659. 10.1016/j.bbi.2020.02.009; 32097763 PMC7126810

[52] Chen, Y. H., Feng, H. L., Jeng, S. S. (2018). Zinc supplementation stimulates red blood cell formation in rats. International Journal of Molecular Sciences*,* 19 *(*9*),* 2824. 10.3390/ijms19092824; 30231592 PMC6165144

[53] Kersemans, V., Cornelissen, B., Allen, P. D., Beech, J. S., Smart, S. C. (2013). MRI was used to measure subcutaneous tumor volume in awake, manually restrained mice. Journal of Magnetic Resonance Imaging*,* 37 *(*6*),* 1499–1504. 10.1002/jmri.23829. 23023925

[54] Gieryng, A., Pszczolkowska, D., Bocian, K., Dabrowski, M., Rajan, W. D. et al. (2017). The immune microenvironment of experimental rat C6 gliomas resembles that of human glioblastomas. Scientific Reports*,* 7*,* 17556. 10.1038/s41598-017-17752-w; 29242629 PMC5730558

[55] Menezes, A., dos Reis, G. H., Oliveira-Nunes, M. C., Mariath, F., Cabanel, M. et al. (2019). Live cell imaging supports a key role for histone deacetylase as a molecular target during glioblastoma malignancy downgrade through tumor competence modulation. Journal of Oncology*,* 2019*,* 1–16. 10.1155/2019/9043675; 31531023 PMC6720048

[56] Lee, D. H., Ryu, H. W., Won, H. R., Kwon, S. H. (2017). Advances in epigenetic glioblastoma therapy. Oncotarget*,* 8 *(*11*),* 18577–18589. 10.18632/oncotarget.14612; 28099914 PMC5392350

[57] Poff, A. M., Ward, N., Seyfried, T. N., Arnold, P., D’Agostino, D. P. (2015). Nontoxic metabolic management of metastatic cancer in VM mice: Novel combination of ketogenic diet, ketone supplementation, and hyperbaric oxygen therapy. PLoS One*,* 10*,* 1–21. 10.1371/journal.pone.0127407 PMC4464523 26061868

[58] Morscher, R. J., Aminzadeh-Gohari, S., Hauser-Kronberger, C., Feichtinger, R. G., Sperl, W. et al. (2016). Combination of metronomic cyclophosphamide and dietary intervention inhibits neuroblastoma growth in a CD1-nu mouse model. Oncotarget*,* 7 *(*13*),* 17060–17073. 10.18632/oncotarget.7929; 26959744 PMC4941371

[59] Aggarwal., A., Yuan, Z., Barletta, J. A., Lorch, J. H., Nehs, M. A. (2020). Ketogenic diet combined with the antioxidant N-acetylcysteine inhibits tumor growth in a mouse model of anaplastic thyroid cancer. Surgery*,* 167 *(*1*),* 87–93. 10.1016/j.surg.2019.06.042; 31521320

[60] Fadaka, A., Ajiboye, B., Ojo, O., Adewala, O., Olayide, I. et al. (2017). Biology of glucose metabolism in cancer cells. Journal of Oncological Sciences*,* 3 *(*2*),* 45–51. 10.1016/j.jons.2017.06.002

[61] Klement, R. J. (2019). The emerging role of ketogenic diets in cancer treatment. Current Opinion in Clinical Nutrition and Metabolic Care*,* 22 *(*2*),* 129–134. 10.1097/MCO.0000000000000540; 30531479

